# Mutations in Splicing Factor Genes Are a Major Cause of Autosomal Dominant Retinitis Pigmentosa in Belgian Families

**DOI:** 10.1371/journal.pone.0170038

**Published:** 2017-01-11

**Authors:** Caroline Van Cauwenbergh, Frauke Coppieters, Dimitri Roels, Sarah De Jaegere, Helena Flipts, Julie De Zaeytijd, Sophie Walraedt, Charlotte Claes, Erik Fransen, Guy Van Camp, Fanny Depasse, Ingele Casteels, Thomy de Ravel, Bart P. Leroy, Elfride De Baere

**Affiliations:** 1 Center for Medical Genetics Ghent, Ghent University and Ghent University Hospital, Ghent, Belgium; 2 Department of Ophthalmology, Ghent University and Ghent University Hospital, Ghent, Belgium; 3 Center for Human Genetics, University Hospitals Leuven, Louvain, Belgium; 4 Center for Medical Genetics Antwerp, Antwerp University, Antwerp, Belgium; 5 Department of Ophthalmology, Hôpital Erasme-ULB, Brussels, Belgium; 6 Department of Ophthalmology, University Hospitals Leuven, Louvain, Belgium; 7 Division of Ophthalmology & Center for Cellular & Molecular Therapy, The Children’s Hospital of Philadelphia, Philadelphia, Pennsylvania, United States of America; University of Florida, UNITED STATES

## Abstract

**Purpose:**

Autosomal dominant retinitis pigmentosa (adRP) is characterized by an extensive genetic heterogeneity, implicating 27 genes, which account for 50 to 70% of cases. Here 86 Belgian probands with possible adRP underwent genetic testing to unravel the molecular basis and to assess the contribution of the genes underlying their condition.

**Methods:**

Mutation detection methods evolved over the past ten years, including mutation specific methods (APEX chip analysis), linkage analysis, gene panel analysis (Sanger sequencing, targeted next-generation sequencing or whole exome sequencing), high-resolution copy number screening (customized microarray-based comparative genomic hybridization). Identified variants were classified following American College of Medical Genetics and Genomics (ACMG) recommendations.

**Results:**

Molecular genetic screening revealed mutations in 48/86 cases (56%). In total, 17 novel pathogenic mutations were identified: four missense mutations in *RHO*, five frameshift mutations in *RP1*, six mutations in genes encoding spliceosome components (*SNRNP200*, *PRPF8*, and *PRPF31*), one frameshift mutation in *PRPH2*, and one frameshift mutation in *TOPORS*. The proportion of *RHO* mutations in our cohort (14%) is higher than reported in a French adRP population (10.3%), but lower than reported elsewhere (16.5–30%). The prevalence of *RP1* mutations (10.5%) is comparable to other populations (3.5%-10%). The mutation frequency in genes encoding splicing factors is unexpectedly high (altogether 19.8%), with *PRPF31* the second most prevalent mutated gene (10.5%). *PRPH2* mutations were found in 4.7% of the Belgian cohort. Two families (2.3%) have the recurrent *NR2E3* mutation p.(Gly56Arg). The prevalence of the recurrent *PROM1* mutation p.(Arg373Cys) was higher than anticipated (3.5%).

**Conclusions:**

Overall, we identified mutations in 48 of 86 Belgian adRP cases (56%), with the highest prevalence in *RHO* (14%), *RP1* (10.5%) and *PRPF31* (10.5%). Finally, we expanded the molecular spectrum of *PRPH2*, *PRPF8*, *RHO*, *RP1*, *SNRNP200*, and *TOPORS*-associated adRP by the identification of 17 novel mutations.

## Introduction

Retinitis pigmentosa (RP) represents the most frequent subtype of inherited dystrophies (iRDs) caused by progressive loss of photoreceptors. The first symptoms in adolescence or early adulthood include night blindness, followed by progressive loss of peripheral visual field in daylight, and eventually culminating in severe visual impairment or blindness after several decades. All modes of Mendelian inheritance can be found in RP, with autosomal dominant (ad) inheritance accounting for 30% to 40% of RP, depending on the population studied [1]. While to date 27 genes and one locus have been identified (RetNet, https://sph.uth.edu/retnet, November 2016), they can only explain 50% to 70% of adRP cases [2,3]. During the last decade, mutation identification studies have shifted from screening of a set of known mutations (e.g. using APEX chip, www.asperbio.com) to targeted next-generation sequencing (NGS) of the coding region of large gene panels [4,5], whole exome sequencing (WES) [6] and whole genome sequencing [7].

Here, we report molecular findings in 86 Belgian families with adRP, identifying 48 mutations, including 17 novel mutations in *PRPH2*, *PRPF8*, *RHO*, *RP1*, *SNRNP200*, and *TOPORS*.

## Materials and Methods

### Patient cohort

This study was conducted following the tenets of the Declaration of Helsinki and ethical approval was given by the local ethics committee. All Belgian patients were enrolled in a clinical context. We followed the standard routine practice and obtained verbal consent by the referring physician in agreement with the Belgian legislation. RP was diagnosed based on measurement of best-corrected visual acuity, slit-lamp biomicroscopy, and fundus photography. Additional tests included Goldmann kinetic perimetry, electroretinography, spectral domain optical coherence tomography, and autofluorescence imaging.

Genomic DNA (gDNA) was extracted from leukocytes using the QIAamp DNA mini kit (Qiagen, Antwerp, Belgium), the Gentra Puregene Cell kit (Qiagen, Antwerp, Belgium), or the ReliaPrep Large Volume HT gDNA Isolation System (Promega, Leiden, The Netherlands) according to the manufacturer’s protocols.

Our overall cohort consists of 86 unrelated Belgian index patients, collected over the past ten years, originating from families with at least two affected generations, of which n = 49 with more than two generations, and n = 38 with male-to-male transmission.

### APEX chip testing

The commercially available arrayed primer extension microarray chip (APEX chip, Asper Biotech, Tartu, Estonia) was a standard test between November 2007 and December 2012. The initial APEX chip version (v. 2.0) was used from November 2007 to November 2008 and included 353 mutations in 13 adRP genes. This chip was regularly updated with new mutations. The latest version (v. 3.0) included 414 mutations in 16 genes.

### Genome-wide linkage analysis and targeted next-generation sequencing (NGS)

Seven families underwent genome-wide linkage analysis. Inclusion criteria were three or more generations of affected members, male-to-male transmission and access to at least six samples from healthy and affected family members. Genome-wide SNP chip genotyping (HumanCytoSNP-12 BeadChip, Illumina) and multipoint linkage analysis (Merlin, dominant model, 95% penetrance, disease allele frequency of 0.0001) was performed on all available family members. A customized microsatellite panel working under uniform PCR conditions was designed for segregation analysis of known adRP genes and the RP63 locus on chromosome 6q23 (S1 Table). Data analysis was performed using the GeneMapper software (Applied Biosystems). Next, adRP genes were selected for downstream NGS analysis based on their presence in loci with the highest LOD-scores.

### PCR and Sanger sequencing

All index patients collected between 2006 and 2012 were tested for mutations in the exons and intron-exon boundaries of the four most prevalent adRP genes (*RP1*, *RHO*, *PRPH2*, *PRPF31*). Mutations found by other techniques were confirmed using PCR and Sanger sequencing (https://pxlence.com; primers available on request).

All index patients were screened for the recurrent *NR2E3* mutation c.166G>A p.(Gly56Arg) and the recurrent *PROM1* mutation c.1117C>T p.(Arg373Cys).

### Targeted NGS

Starting from 2012, a targeted NGS panel was introduced using a flexible protocol, consisting of singleplex PCR followed by NexteraXT library preparation and sequencing on a MiSeq instrument [8]. The CLC Genomics Workbench v.6 (Qiagen) was employed for read mapping against the hg19 human reference genome and variant calling. To date, our diagnostic panel consists of ten adRP genes (*CRX*, *PRPF6*, *PRPF8*, *PRPF31*, *PRPH2*, *RDH12*, *RHO*, *RPE65*, *RP1*, *SNRNP200*).

### Whole exome sequencing (WES)

Targeted WES was implemented in 2015. Whole exome enrichment was performed using the SureSelectXT human All Exon V5 enrichment kit (Agilent) followed by sequencing on a NextSeq500 (Illumina). The CLC Genomics Workbench (v. 7.5.4, Qiagen) was employed for read mapping against the hg19 human reference genome, and variant calling. Annotation and filtering of variants was done using an in-house developed strategy. Based on variant allele frequency, variants were categorized as heterozygous (20%–70%) or homozygous (>70%). Variant filtering was performed against a list of RetNet genes (gene panel v. 4, 226 genes).

### ArrayCGH platform

A customized array comparative genomic hybridization platform (arrayCGH), called arrEYE, was used for high-resolution copy number variant analysis of 106 known and 60 candidate genes for iRD and 196 retina-expressed non-coding RNAs (ncRNAs) [9]. The data was processed and analyzed with the ViVar software (http://www.cmgg.be/vivar/).

### Variant interpretation

The functional impact of sequence variants was assessed based on the outcome of *in silico* predictions performed in Alamut Visual (v. 2.7) or Alamut HT/Alamut Batch (for WES data), including splice prediction tools (SpliceSiteFinder-like, MaxEntScan, NNSPLICE), and missense prediction tools (SIFT, Polyphen-2, Align GVGD and Mutation Taster), assessment of physicochemical distance (Grantham score calculation), evolutionary conservation, location in protein domains, presence in dbSNP build 145 (http://www.ncbi.nlm.nih.gov/SNP/), Exome Variant Server from the NHLBI Exome Sequencing Project (ESP, http://evs.gs.washington.edu/EVS/), ExAC (http://exac.broadinstitute.org) and gnomAD (http://gnomad.broadinstitute.org) [10]. All variants were verified in the public version of the Human Gene Mutation Database (http://www.hgmd.cf.ac.uk/ac/index.php) combined with a thorough literature search. The recent ACMG guidelines were applied for classification of the sequence variants [11]. The maximum tolerated reference allele count was calculated for all variants present in public databases (ExAC, gnomAD) using an online calculator (https://jamesware.shinyapps.io/alleleFrequencyApp/) (S2 Table) [12]. HGVS mutation nomenclature was used, with the A of the initiation codon ATG as +1 (http://www.hgvs.org/mutnomen).

## Results and Discussion

### Mutation detection rate and prevalence of mutations

To date, mutations in 27 adRP genes have been reported in adRP [RetNet, November 2016]. Depending on the technology used, the mutation detection varies from 50 to 70% [2,3]. We applied several screening methods over the past ten years (Table 1). Screening of known mutations (APEX chip) revealed mutations in ten cases. In 2011 a combined approach of genome-wide linkage analysis and targeted NGS on a selected set of seven families identified mutations in known adRP genes in all seven families, with all genes located in regions with the highest LOD score. A retrospective screen of the four most prevalent adRP genes (*RHO*, *RP1*, *PRPH2*, *PRPF31*), the recurrent *NR2E3* p.(Gly56Arg) and *PROM1* p.(Arg373Cys) mutations was performed in all 86 index cases initially using Sanger sequencing and subsequently using targeted next-generation sequencing (NGS) on MiSeq. In parallel to targeted NGS of an extended adRP panel, targeted WES (based on integrated variant annotation and filtering of RetNet genes) was introduced. Together, these targeted sequencing approaches revealed mutations in 31 cases (Sanger sequencing n = 16; targeted NGS on MiSeq n = 12; WES n = 3). These molecular screening methods were recently complemented by copy number variant (CNV) analysis using a high-resolution customized array called arrEYE, containing probes for the exonic and entire intronic regions of 106 known iRD genes, including all 27 adRP genes. No copy number variations were identified in the screened adRP cohort so far [9].

**Table 1:** Mutations identified in the Belgian cohort.

Gene	Exon	cDNA	Protein	Method	FAM ID	Segr.	ACMG	A. GVGD	SIFT	PolyP.	MT	Gran.	NT cons.	AA cons.	Splicing	EXAC	GnomAD (beta)	ESP	Ref
*RHO*	1	c.44A>G	p.(Asn15Ser)	Sanger	FAM_001	NA	Class 5	C0	Delet.	Prob. dam.	D	46	High phyloP:4.97	High,up to Tetraodon	/	NP	**NP**	/	[31]
*RHO*	1	c.265G>C	p.(Gly89Arg)	Sanger	FAM_002	NA	Class 4	C0	Delet.	Poss. dam.	D	125	Weak phyloP:1.74	High,up to Tetraodon	/	NP	**NP**	/	Novel
*RHO*	2	c.403C>T	p.(Arg135Trp)	APEX/Sanger	FAM_003_004	yes	Class 5	C65	Delet.	Prob. dam.	D	101	Weak phyloP:0.45	High,up to Tetraodon	/	NP	NP	/	[32]
*RHO*	3	c.532T>G	p.(Tyr178Asp)	APEX	FAM_005	yes	Class 5	C65	Delet.	Prob. dam.	D	160	High phyloP:4.89	High,up to Tetraodon	/	NP	NP	/	Novel
*RHO*	3	c.563G>A	p.(Gly188Glu)	APEX	FAM_006	NA	Class 5	C65	Delet.	Prob. dam.	D	98	High phyloP:6.02	High,up to Tetraodon	/	NP	0.000396%*	/	[33]
*RHO*	4	c.763_765del	p.(Ile256del)	APEX	FAM_007	yes	Class 5	/	/	/	/	/	/	/	/	NP	NP	/	[34]
*RHO*	4	c.911T>A	p.(Val304Asp)	Sanger	FAM_008	yes	Class 3	C45	Delet.	Prob. dam.	D	152	Mod. phyloP:3.35	High,up to Frog	/	NP	NP	/	Novel
*RHO*	5	c.1028G>A	p.(Ser343Asn)	NGS/NGS	FAM_009_010	yes	Class 5	C45	Delet.	Poss. dam.	D	46	High phyloP:5.61	High,up to Tetraodon	/	NP	NP	/	Novel
*RHO*	5	c.1033G>A	p.(Val345Met)	APEX/APEX	FAM_011_012	NA	Class 5	C15	Delet.	Prob. dam.	D	21	High phyloP:5.61	High,up to Tetraodon	/	NP	NP	/	[35]
*RP1*	4	c.2026del	p.(Ser676Leufs*6)	NGS/NGS	FAM_013_014	NA	Class 5	/	/	/	/	/	/	/	PTC (last exon)	NP	NP	/	Novel
*RP1*	4	c.2029C>T	p.(Arg677*)	NGS	FAM_015	NA	Class 5	/	/	/	/	/	/	/	PTC (last exon)	NP	NP	/	[20]
*RP1*	4	c.2200del	p.(Ser734Valfs*4)	Sanger	FAM_016	NA	Class 5	/	/	/	/	/	/	/	PTC (last exon)	NP	NP	/	Novel
*RP1*	4	c.2245_2248delinsTGAG	p.(Leu749*)	Linkage	FAM_017	yes	Class 5	/	/		/	/	/	/	PTC (last exon)	NP	NP	/	Novel
*RP1*	4	c.2305_2317del	p.(Lys769Phefs*2)	Linkage	FAM_018	yes	Class 5	/	/		/	/	/	/	PTC (last exon)	NP	NP	/	Novel
*RP1*	4	c.2597del	p.(Leu866*)	NGS	FAM_019	NA	Class 5	/	/	/	/	/	/	/	PTC (last exon)	NP	NP	/	Novel
*RP1*	4	c.3157del	p.(Tyr1053Thrfs*4)	Sanger/NGS	FAM_020_021	yes	Class 5	/	/	/	/	/	/	/	PTC (last exon)	0.00165%**	NP	/	[42]
*SNRNP200*	15	c.1981G>T	p.(Val661Leu)	Linkage	FAM_022	yes	Class 4	C0	Delet.	Prob. dam.	D	32	High phyloP:6.18	High,up to Baker's yeast	/	NP	NP	/	Novel
*SNRNP200*	16	c.2041C>T	p.(Arg681Cys)	Linkage	FAM_023	yes	Class 5	C0	Delet.	Prob. dam.	D	180	High phyloP:6.10	High,up to Baker's yeast	/	NP	0.000396%***	/	[47]
*SNRNP200*	16	c.2042G>A	p.(Arg681His)	NGS	FAM_024	NA	Class 5	C0	Delet.	Prob. dam.	D	29	High phyloP:6.10	High,up to Baker's yeast	/	NP	NP	/	[47]
*PRPF8*	42	c.6840C>A	p.(Asn2280Lys)	Linkage	FAM_025	yes	Class 4	C0	Delet.	Prob. dam.	D	94	Mod. phyloP:2.47	High,up to Baker's yeast	/	NP	NP	/	Novel
*PRPF8*	16	c.6912C>G	p.(Phe2304Leu)	APEX/WES	FAM_026_027	NA	Class 5	C0	Delet.	Poss. dam.	D	22	Weak phyloP:1.42	Mod. (cons. 11 species)	/	NP	NP	/	[48]
*PRPF8*	43	c.6964G>T	p.(Glu2322*)	WES	FAM_028	yes	Class 5	/	/	/	/	/	/	/	PTC (last exon)	NP	NP	/	Novel
*PRPF8*	43	c.7006T>C	p.(*2336Argext*41)	APEX	FAM_029	yes	Class 5	/	/		/	/	/	/	Extended RF	NP	NP	/	[28]
*PRPF31*	2	c.34G>T	p.(Glu12*)	Sanger/NGS	FAM_030_031	NA	Class 5	/	/		/	/	/	/	PTC (NMD)	NP	NP	/	Novel
*PRPF31*	3	c.220C>T	p.(Gln74*)	APEX/NGS	FAM_032_033	NA	Class 5	/	/		/	/	/	/	PTC (NMD)	NP	NP	/	[2]
*PRPF31*	Intron 6	c.528-1G>A	p.?	Linkage	FAM_034	yes	Class 5	/	/		/	/	/	/	Loss donor site	NP	NP	/	[26]
*PRPF31*	7	c.541G>T	p.(Glu181*)	Sanger/WES	FAM_035_036	NA	Class 5	/	/		/	/	/	/	PTC (NMD)	NP	NP	/	[49]
*PRPF31*	10	c.978_982del	p.(Lys327Argfs*146)	Sanger	FAM_037	NA	Class 5	/	/		/	/	/	/	PTC (NMD)	NP	NP	/	Novel
*PRPF31*	11	c.1077C>A	p.(Tyr359*)	NGS	FAM_038	NA	Class 5	/	/		/	/	/	/	PTC (NMD)	NP	NP	/	Novel
*PRPH2*	1	c.382_385dup	p.(Thr129Lysfs*49)	Sanger	FAM_039	yes	Class 5	/	/	/	/	/	/	/	PTC (NMD)	NP	NP	/	Novel
*PRPH2*	1	c.424C>T	p.(Arg142Trp)	Sanger	FAM_040	NA	Class 3	C35	Delet.	Prob. dam.	D	101	Weak phyloP:0.37	High,up to Chicken	/	0.00247%^‡^	0.00212%^‡‡^	/	[67]
*PRPH2*	1	c.535T>C	p.(Trp179Arg)	NGS	FAM_041	NA	Class 5	C65	Delet.	Prob. dam.	D	101	High phyloP:5.13	High,up to Tetraodon	/	NP	NP	/	[68]
*PRPH2*	2	c.647C>T	p.(Pro216Leu)	Sanger	FAM_042	NA	Class 4	C0	Delet.	Benign	D	98	Mod. phyloP:4.24	High,up to Tetraodon	/	NP	0.000396%^‡‡‡^	/	[16]
*NR2E3*	2	c.166G>A	p.(Gly56Arg)	Sanger/Sanger	FAM_043_044	yes	Class 5	C15	Delet.	Prob. dam.	/	125	Weak phyloP:9.53	High (up to Fruitfly)	/	NP	NP	/	[73]
*PROM1*	12	c.1117C>T	p.(Arg373Cys)	Linkage/Sanger/Sanger	FAM_045_046_047	yes	Class 5	C0	Delet.	Poss. dam.	D	180	Weak phyloP:0.19	Weak (cons. 16 species)	/	NP	NP	/	[74]
*TOPORS*	3	c.2556_2557del	p.(Glu852Aspfs*20)	APEX	FAM_048	NA	Class 5	/	/	/	/	/	/	/	PTC (last exon)	NP	NP	/	Novel

Refseq transcripts (GRCh37/hg19): *RHO* (NM_000539.3), *RP1* (NM_006269.1), *SNRNP200* (NM_014014.4), *PRPF8* (NM_006445.3), *PRPF31* (NM_006445.3), *PRPH2* (NM_000322.4), *NR2E3* (NM_014249.2), *PROM1* (NM_006017.2), *TOPORS* (NM_005802.4).

American College of Medical Genetics and Genomics (ACMG) classification: class 1, benign; class 2, likely benign; class 3, uncertain significance; class 4, likely pathogenic; class 5, pathogenic.

* 0.000396%; gnomAD allele count: 1/252,388 for all WES alleles; with 1/35,710 alleles for the Latino population.

** 0.00165%; ExAC allele count: 2/121,108 for all WES alleles; with 2/66,658 alleles for the European (Non-Finnish) population.

*** 0.000396%; gnomAD allele count: 1/252,348 for all WES alleles; with 1/112,194 alleles for the European (Non-Finnish) population.

^‡^ 0.00247%; ExAC allele count: 3/121,412 for all WES alleles; with 2/11,578 alleles for the Latino and 1/66,740 for the European (Non-Finnish) population.

^‡‡^ 0.00212%; gnomAD allele count: 6/282,618 for all WES alleles; with 3/36,474 for the Latino and 3/126,764 for the European (Non-Finnish) population.

^‡‡‡^ 0.000396%; gnomAD allele count: 1/252,394 for all WES alleles; with 1/112,228 alleles for the European (Non-Finnish) population.

AA cons., amino acid conservation; APEX, arrayed primer extension microarray chip; D: Disease causing; Delet., deleterious; Gran., Grantham score; Linkage, genome-wide linkage analysis; MT: Mutation Taster; NA, not available; NMD, nonsense mediated decay; NGS, Next-generation sequencing using MiSeq; NT cons., nucleotide conservation; PolyP., PolyPhen-2; Poss. dam., possibly damaging; Prob. dam., probably damaging; PTC: premature termination codon; Sanger, Sanger sequencing; Segr., Segregation.

**Fig 1:**
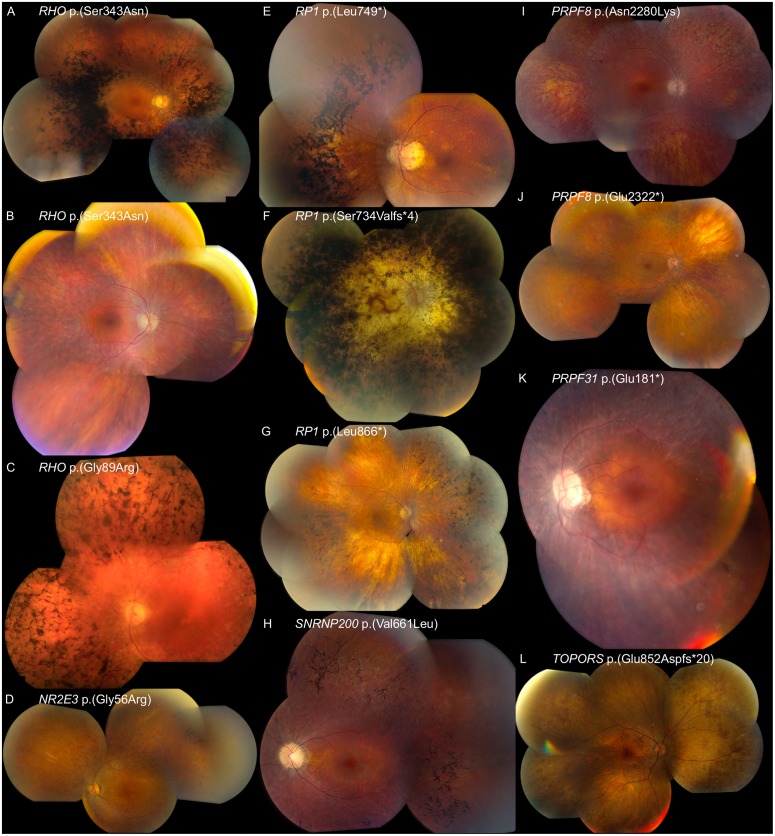
Composite fundus photographs of 12 patients with mutations in *RHO*, *RP1*, *SNRNP200*, *PPRF8*, *PRPF31*, *TOPORS* and *NR2E3* leading to adRP. Overall, the phenotypes shown represent a range of adRP phenotypes varying from milder, classic, to end-stage RP. (A) Age 55 years (FAM_009), *RHO* mutation, c.1028G>A p.(Ser343Asn) (novel). A classic RP phenotype, including good macular preservation, attenuated retinal vasculature, outer retinal atrophy and predominantly spicular intraretinal pigment migration in the midperiphery. (B) Age 54 years (FAM_010), *RHO* mutation, c.1028G>A p.(Ser343Asn) (novel). Milder phenotype compared to A. Diffuse outer retinal atrophy in the periphery with good macular preservation. Notice the absence of intraretinal pigment migration. (C) Age 55 years (FAM_002), *RHO* mutation, c.265G>C p.(Gly89Arg) (novel). End-stage RP with macular atrophy, attenuated retinal vasculature and diffuse intraretinal pigment migration in the midperiphery. (D) Age 53 years (FAM_043), recurrent *NR2E3* mutation, c.166G>A p.(Gly56Arg). Outer retinal atrophy with mild intraretinal pigment migration in the periphery and perifoveal outer retinal atrophy. (E) Age 51 years (FAM_017), *RP1* mutation, c.2245_2248delinsTGAG p.(Leu749*) (novel). A classic RP phenotype, similar to the description of panel A. (F) Age 72 years (FAM_016), *RP1* mutation, c.2200del p.(Ser734Valfs*4) (novel). End-stage RP with complete outer retinal atrophy and intraretinal pigment migration including periphery and macula. (G) Age 72 years (FAM_019), *RP1* mutation, c.2597del p.(Leu866*) (novel). Typical yellowish hue due to outer retinal atrophy with intraretinal pigment migration in the periphery and macular preservation. (H) Age 30 years (FAM_022), *SNRNP200* mutation, c.1981G>T p.(Val661Leu) (novel). Outer retinal atrophy with spicular intraretinal pigment migration, most pronounced in the retinal midperiphery. (I) Age 38 years (FAM_025), *PRPF8* mutation, c.6840C>A p.(Asn2280Lys) (novel). Outer retinal atrophy with intraretinal pigment migration of the spicular type in the midperiphery and a good macular preservation. (J) Age 50 years (FAM_028), *PRPF8* mutation, c.6964G>T p.(Glu2322*) (novel). Mild outer retinal atrophy in the periphery with macular preservation, normal retinal vasculature and a normal optic disc. (K) 53 years (FAM_035), *PRPF31* mutation, c.541G>T p.(Glu181*). Outer retinal atrophy with macular preservation. (L) 51 years (FAM_048), *TOPORS* mutation, c.2556_2557del p.(Glu852Aspfs*20) (novel). Pigment epithelium alterations with white dots in the retinal periphery. Notice absence of intraretinal pigment migration and presence of perifoveal atrophy.

Overall molecular genetic screening revealed mutations in 48 out of 86 cases (56%), 36 of which are distinct mutations. Since only a minority of patients underwent RetNet-based filtering of WES data, this detection rate will probably increase in the coming years. Seventeen mutations are novel and are discussed in this paper (Table 1). Representative fundus pictures of 12 patients with novel mutations in adRP genes are shown in Fig 1.

**Fig 2:**
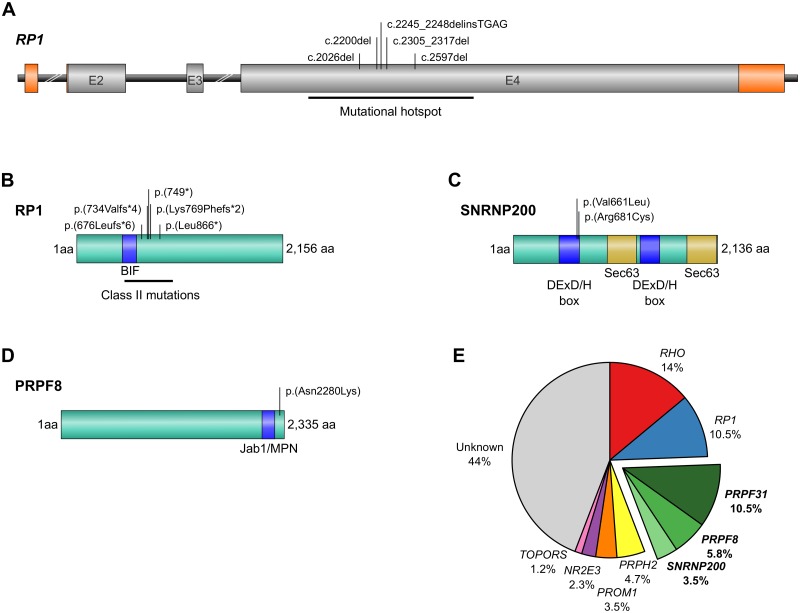
Schematic representation of novel mutations and prevalence of causal mutations in adRP genes. (A-D): Schematic representation of the novel mutations identified in this study. (A) *RP1* gene. The five mutations are located within the mutational hotspot (nucleotides 1490–3216), indicated with a black horizontal line. E = exon. Grey rectangles are coding regions and orange rectangles are 5’ untranslated region (5’ UTR) and 3’ UTR. (B) RP1 protein. Both truncating mutations identified in this study belong to Class II mutations (amino acids 500–1053), indicated with a black line. The *Drosophila melanogaster* (BIF) domain (amino acids 486–635) is depicted as a blue rectangle. aa = amino acid. (C) SNRNP200 protein. The two novel mutations identified in this study are both located within the first DExD/H box helicase-like domain (amino acids 477–690). Both the first and the second (amino acids 1324–1528) DExD/H box helicase domains are represented as blue rectangles. Both Sec63-like domains (amino acids 981–1286 and 1812–2124) are indicated as golden rectangles. aa = amino acids. (D) PRPF8 protein. The novel mutation identified here is located within the highly conserved region C-terminal to the Jab1/MPN domain (amino acids 2099–2233), depicted as a blue rectangle. aa = amino acid. (E) Prevalence of causative mutations in adRP genes in a Belgian adRP cohort. The ‘unknown’ part may include new disease genes and mutation mechanisms as well as known disease genes not screened in the course of this study.

The prevalence in our population can only be determined for the four most common disease genes (*RHO*, *RP1*, *PRPH2*, *PRPF31*) and the recurrent *PROM1* and *NR2E3* mutations that were screened in the entire cohort. Mutations in the *rhodopsin* (*RHO*, NM_000539.3, MIM# 613731) gene are the most common cause of adRP. The prevalence of *RHO* mutations in the Belgian adRP population is approximately 14%. This is higher than the prevalence in France (10.3%), but lower compared to other populations (European cohorts: 16.5%-30%, American cohorts: approximately 30%) [2,13–19]. Mutations in the *retinitis pigmentosa 1* gene (*RP1*, NM_006269.1, MIM# 603937) account for 10.5% of the Belgian cohort, which is higher than the prevalence in Spain (3.5%), Italy (5%), and France (5.3%), but closer to the prevalence in other cohorts in US (7.7%) and the United Kingdom (8%-10%) [2,17,20–25]. *PRPF31* mutations account for 10.5% of adRP in the Belgian cohort. This is higher than the previously reported prevalence in US (5.5%), the United Kingdom (5%) and France (6.7%) [2,26–27]. The prevalence of mutations in genes encoding three splicing factors (*PRPF8*, *PRPF31* and *SNRNP200*) is high (altogether 19.8%). Together, these mutations represent the most common cause of adRP in the Belgian adRP cohort [2,28]. The prevalence of *peripherin 2* (*PRPH2*) mutations in adRP widely varies depending on the origin, from 0% (Mexican cohort) up to 10.3% (French cohort) [2,29–30]. Here, mutations in *PRPH2* account for 4.7%. The prevalence of the recurrent *PROM1* mutation p.(Arg373Cys) is higher (3.5%) than anticipated in adRP. Two families have the recurrent *NR2E3* mutation p.(Gly56Arg), accounting for 2.3% (Fig 2).

## Sequence and Copy Number Variations

### *RHO* mutations

We identified nine distinct *RHO* (NM_000539.3, MIM# 613731) mutations, four of which are novel: c.265G>C p.(Gly89Arg), c.532T>G p.(Tyr178Asp), c.911T>A p.(Val304Asp) and c.1028G>A p.(Ser343Asn) (n = 2), (Table 1) [31–35]. All four missense substitutions change a highly conserved amino acid and are predicted to be deleterious. For the missense variant c.532T>G p.(Tyr178Asp), different changes of the same amino acid p.(Tyr178Asn) and p.(Tyr178Cys) have been reported [32,36]. Out of a total of 12 identified mutations, the two novel missense substitutions p.(Val304Asp) and p.(Ser343Asn) were the only variants not present in previously affected amino acids of the RHO protein (http://www.retina-international.org/files/sci-news/rhomut.htm). Interestingly, the p.(Ser343Asn) substitution disrupts the phosphorylation site closest to the C-terminus and is known to play a crucial role in promoting the binding of the rod-specific arrestin to rhodopsin [37,38]. Moreover, this substitution resides within a hot spot region between p.(Thr340) and p.(Pro347), with mutations reported in all consecutive amino acids except for p.(Ser343). The p.(Val304Asp) variant is located in the seventh transmembrane segment within the highly conserved NPxxY-motif (Asn302/Pro303/Val304/Ille305/Tyr306). A key function of this motif is to mediate several inter-helical interactions that might have a potentially stabilizing role to maintain the ground state structure of RHO [39–41]. The pathogenicity of p.(Val304Asp) is uncertain based on its position within the motif. The most prevalent European *RHO* mutation, p.(Pro347Leu), was not observed in our cohort [19].

### *RP1* mutations

Seven distinct pathogenic variants were found in the *RP1* gene (NM_006269.1, MIM# 603937), five of which are novel (Table 1) [20,42]. A heterozygous indel mutation c.2245_2248delinsTGAG, replacing the nucleotides CTCA by their reverse complement TGAG p.(Leu749*), and four heterozygous deletions: c.2305_2317del p.(Lys769Phefs*2), c.2026del p.(Ser676Leufs*6) (n = 2); c.2200del p.(Ser734Valfs*4); c.2597del p.(Leu866*) were found. These five novel mutations create a premature termination codon (PTC) in the last exon that is predicted to escape nonsense-mediated decay (NMD) and to lead to a truncated protein. This is in line with the majority of *RP1* alleles that generate PTCs located in a mutational hotspot region (c.1490-c.3216) within the last exon [25]. Indeed, all seven mutations identified here are located within this hotspot region (Fig 2). The numerous deletions and insertions can be explained by the multiple A nucleotides flanking the mutation sites, possibly causing slipped strand mispairing during replication [43]. Chen et al. proposed four classes of *RP1* mutations [44]. Truncations located between amino acid 500 and 1053 within the last exon are NMD-insensitive and belong to ‘Class II’ mutations, making up the majority of PTC mutations. A loss of the C-terminal half to one third of the RP1 protein may have a deleterious effect through the exposure of the *Drosophila melanogaster* bifocal (BIF) domain (amino acids 486–635) (Fig 2). This eventually results in a potential dominant negative effect rather than haploinsufficiency as an underlying mechanism [45].

### Mutations in splicing factor genes *SNRNP200*, *PRPF8*, *PRPF31*

Today, seven ubiquitously expressed adRP genes involved in nuclear pre-messenger RNA (pre-mRNA) splicing have been described (RetNet). Six of them encode components of the U4/U6-U5 triple small nuclear ribonucleoprotein (tri-snRNP) complex of the spliceosome, highlighting its important role in adRP pathogenesis [46]. Overall, mutations in splicing factor genes are the second most common cause of adRP [2]. We identified heterozygous mutations in adRP splicing genes (*SNRNP200*, *PRPF8*, *PRPF31*) in 17 probands (Table 1) [2,26,28,47–49].

A novel heterozygous missense variant was revealed in *SNRNP200* (NM_014014.4, MIM# 601664), c.1981G>T p.(Val661Leu). The Val residue is highly conserved up to Baker’s yeast and the mutation is predicted to be deleterious (Table 1). The splicing factor *SNRNP200* encodes BRR2, a stable component of the U5 snRNP that is essential for the unwinding of the U4/U6 and U2/U6 snRNAs [50,51]. BRR2 interacts extensively with the U5-specific protein PRPF8 [52]. The *SNRNP200* mutations found in Belgian cases are located in the first of two DExD/H box helicase-like domains, in line with most previously described mutations (Fig 2) [53–56].

We identified a novel heterozygous missense mutation in *PRPF8* (NM_006445.3, MIM# 600059), c.6840C>A p.(Asn2280Lys). This variant alters a highly conserved amino acid (up to Baker’s yeast) and is predicted to have a possible effect on the protein structure or function. A second, novel *PRPF8* nonsense mutation c.6964G>T p.(Glu2322*) was found (Table 1), introducing a PTC in the last exon, predicted to escape NMD and to lead to a truncated protein. The U5 snRNP protein PRPF8 is crucial for the formation of the catalytic center in the spliceosome and interacts via its C-terminus with the DExD/H domain, suggesting that mutations might affect the PRPF8-BBR2 interaction [53]. Indeed, *PRPF8* mutations that lead to adRP cluster within the highly conserved region C-terminal to the Jab1/MPN domain. The three mutations found here are located within the same C-terminal domain (Fig 2). In yeast this specific domain forms a complex with BRR2 and stimulates its helicase activity [57–60].

*PRPF31* (NM_015629.3, MIM# 600138) encodes an U4/U6-specific protein that interacts with the U4 snRNA and facilitates the formation of tri-snRNP by physically tethering U4/U6 and U5 snRNPs [61–63]. A novel heterozygous *PRPF31* nonsense substitution was identified in two unrelated probands, c.34G>T p.(Glu12*), likely subjecting the transcript to NMD. In total, nine patients were found to have mutations in *PRPF31*, seven nonsense mutations, one out-of-frame deletion, and one splice-site mutation (Table 1). The greater part of *PRPF31* mutations described in literature are (large) deletions, insertions, duplications, nonsense and splice-site mutations leading to haploinsufficiency [64]. No large rearrangements of *PRPF31* were identified in the studied cohort, however [9]. Variable expression or non-penetrance have been reported in adRP families with mutations in the *PRPF31* gene (for review see Rose and Bhattacharya, 2016) [65]. Two out of nine Belgian families exhibited apparent non-penetrance. The c.528-1G>A mutation (FAM_034) segregates with the disease in the family, notably one carrier family member, the sib of the index patient exhibited no clinical signs. In a three generation family (FAM_035, c.541G>T p.[Glu181*]), two obligate carrier females of the second generation have both affected children, but do not show clinical signs.

### *TOPORS* mutation

We identified a novel deletion, c.2556_2557del p.(Glu852Aspfs*20), in the *topoisomerase I-binding arginine-serine rich* gene (*TOPORS*, NM_005802.4, MIM# 609923) (Table 1). The majority of the reported *TOPORS* mutations are located within the same region of the last exon, lead to a PTC and are predicted to escape NMD. The lack of a truncated protein in patients’ lymphoblastoid cells however indicates an unstable mutant protein, suggesting haploinsufficiency, rather than a dominant negative effect as a disease mechanism [66].

### *PRPH2* mutations

Three out of four variants found in the *PRPH2* gene have previously been described in adRP patients (Table 1) [16,67,68]. A novel out-of-frame duplication was found, c.382_382dup p.(Thr129Lysfs*49), likely subjecting the mRNA to NMD. The *PRPH2* gene encodes a transmembrane glycoprotein located at the rim regions of photoreceptor outer segment discs [69]. It forms a homo-oligomeric structure that subsequently assembles into homo-tetramers, or forms hetero-tetrameric complexes with its paralogous protein, rod outer segment protein 1 (ROM1) [70]. These protein structures have an important role in photoreceptor disc morphogenesis and stabilization [71]. The majority of *PRPH2* mutations are sequence variants. Different mechanisms, including aberrant mRNA splicing, protein mislocalization, and protein degradation may cause a reduced expression of the protein in the rod outer segment [72].

### Recurrent *NR2E3* and *PROM1* mutations

The recurrent *nuclear receptor subfamily 2*, *group E*, *member 3* (*NR2E3*, NM_014249.2, MIM# 611131) mutation p.(Gly56Arg) was found in two index patients (Table 1) [73]. In addition, the recurrent *prominin 1* (*PROM1*, NM_006017.2, MIM# 608051) mutation p.(Arg373Cys) was found in three cases (Table 1) [74]. Patients with the recurrent *PROM1* mutation are known to have phenotypes ranging from isolated macular dystrophy, rod dystrophy, rod-cone dystrophy and cone-rod dystrophy. Most reported cases present with a bull’s eye maculopathy [74–75]. The index patients in our cohort were referred with a tentative diagnosis of adRP and adRP with macular involvement. A reclassification may be required based on a detailed clinical examination of family members. Since extraocular phenotypes have been described in some patients with the recurrent *PROM1* mutation, this finding may have clinical implications [76].

### Copy number variants

In 2016 we developed and implemented arrEYE, a microarray-based platform for high-resolution copy number analysis in iRD [9]. Using this approach we previously identified a novel heterozygous deletion of exons 7 and 8 of the *Heparan-Alpha-Glucosaminide N-Acetyltransferase* (*HGSNAT*, NM_152419.2, MIM# 616544) gene: c.634-408_820+338delinsAGAATATG, p.(Glu212Glyfs*2) in a simplex RP patient. A second variant p.(Arg615Thr) was identified on the other allele [9]. No disease-causing CNVs were found in the adRP cohort studied so far.

### Classification of variants

Variants were classified following the ACMG standards and guidelines, categorizing them in one of five classes (pathogenic, likely pathogenic, uncertain significance, likely benign and benign) [11]. For the majority of variants, this categorization is in line with former classifications based on *in silico* predictions and literature searches. The classification was debatable for several variants that are listed in ExAC and gnomAD (beta, December 2016). Recently, Sharon et al. highlighted the importance of population frequency thresholds for the filtering and classification of variants found with WES. It was estimated that the allele frequency of a true adRP disease-causing variant should be lower than 1 in 100,000 (<0.001%), taking into account the heterogeneous nature of the disease, rare incidences of reduced penetrance and undiagnosed individuals mistaken as controls [77]. A statistical framework for a frequency-based filtering was presented by Whiffin et al. (2016; online calculator; https://jamesware.shinyapps.io/alleleFrequencyApp/) [12]. These calculations estimate the maximum tolerated allelic count for a variant in a reference dataset (e.g. ExAC, gnomAD), i.e. a threshold for assessing whether a variant is to commonly present in a reference dataset to be disease causing. The calculation is based on several parameters, including inheritance, disease prevalence, maximum allelic contribution, penetrance and number of screened reference alleles.

The disease prevalence of RP is one in 5000, with about 30% to 40% having a dominant inheritance [1,77]. We applied a (maximum) disease frequency of 1 in 12,500 for adRP. Since only rather small populations were screened for all known adRP genes, and the contribution of each individual mutation (i.e. allelic heterogeneity) is not well known, we assumed that the maximum contribution of each gene (i.e. the prevalence of the disease gene) is the maximum possible allelic contribution. This is an overestimation since adRP is not only characterized by locus heterogeneity, but also by allelic heterogeneity, whereby most variants within a gene only account for a small percentage of cases. Although most alleles are fully penetrant, non-penetrance has been reported. Calculations were made for a variant penetrance of 1, 0.95 and 0.5. The predicted maximum allele count was calculated for all genes with variants present in ExAC or gnomAD (S2 Table). Only one variant exceeded the predicted maximum allele count in the reference databases. The variant, c.424C>T p.(Arg142Trp) in *PRPH2*, is predicted to be deleterious by several *in silico* predictions and has been reported as pathogenic multiple times (also known as R142W), which would qualify it as likely pathogenic [78]. However, the minor allele frequency (MAF) of 0.0021% (gnomAD: present in 6 out of 282,618 alleles) exceeds the expected allele frequency of a dominant mutation in the general population (Table 1) and the allele count is above the threshold (S2 Table). Following this reasoning, this variant should be reclassified as variant of uncertain significance. Moreover, the p.(Arg142Trp) variant was reported in an autosomal recessive RP family with a homozygous pathogenic *PDE6B* mutation. The *PRPH2* variant might explain the more severe phenotype seen in the individual with variants in both disease genes [79]. The p.(Arg142Trp) variant is frequently reported in patients with Central Areolar Choroidal Dystrophy (CACD) (29 out of 60) [78], with a mean age of onset of 46 years [80].

The variant allele counts in the reference datasets for the remainder mutations did not exceed the maximum tolerated allele count (S2 Table). It cannot be excluded that any of the individuals in ExAC and gnomAD with mutations in adRP genes are too young to express the gene-associated adRP or display non-penetrance or a minimal expression.

Altogether, this illustrates that weighting the MAF and the maximum tolerated allele count of variants in genomic databases as a parameter for variant classification cannot be done in an absolute way in the context of dominant diseases with a later age of onset, as variant databases of supposedly control individuals do not contain information on the age of individuals or on phenotypes.

### General conclusion

To summarize, this is the first comprehensive molecular genetic study on adRP-causing mutations in a Belgian cohort of 86 patients. We obtained a molecular diagnosis of adRP in 48 out of 86 cases (56%), with the highest mutation prevalences in *RHO* (14%), *RP1* (10.5%) and *PRPF31* (10.5%). A striking observation is that mutations in splicing factor genes represent the most common cause of adRP in the Belgian cohort (19.8%). Finally, we identified 17 novel mutations in the *RP1*, *RHO*, *PRPH2*, *PRPF31*, *PRPF8*, *SNRNP200*, and *TOPORS* genes, thereby expanding their molecular spectrum. Classification of variants following ACMG guidelines allows a systematic categorization although variant allele frequencies and allele count in public genomic databases should be assessed with caution.

## Supporting Information

S1 Table AdRP microsatellite panel. Three to four pairs of primers were designed for microsatellites flanking an adRP gene within a distance of one to two megabases (Mbs) up- or downstream (Primer3plus; http://www.bioinformatics.nl/cgi-bin/primer3plus/primer3plus.cgi). Six primer pairs were designed for the RP63 locus on chromosome 6q23, with adjacent primers less than one Mb apart. Each forward primer is tagged with a M13-tail. Sequence of the M13-tail: 5’-cacgacgttgtaaaacgac-3’.
(XLSX)

S2 Table Calculation of maximum tolerated allele count. The maximum tolerated allele count was computed using an online calculator (https://jamesware.shinyapps.io/alleleFrequencyApp/). The allele count represents the count of each variant in ExAC or gnomAD for the entire studied population (indicated as ‘all’) and for the individual population groups in which the variant was found. The reference allele number is the total allele count screened in the reference population(s). Inheritance is monoallelic. The prevalence was calculated based on the prevalence of RP (1/5000), with about 30% to 40% having a dominant inheritance. We assumed that the maximum possible allelic contribution (maximum allelic heterogeneity) is the maximum genetic contribution (as described in Whiffin et al. for genes with a less characterized allelic heterogeneity). A range was taken for the maximum allelic heterogeneity; with minimum and maximum value being the in literature reported minimum and maximum contribution of a gene in adRP. Calculations were also made for the prevalence in the Belgian adRP population (B). Since non-penetrance has been described for several genes, we assumed three different penetrance values (1, 0.95 and 0.50). Maximum tolerated ref. AC = Maximum tolerated reference allele count. Blue shaded cells: Values needed for calculation of the maximum tolerated reference allele count. Orange shaded cells: variant counts that exceeded the maximum tolerated reference allele count [12].
(XLSX)
